# *Gymnema sylvestre* as a Multi-Target Antidiabetic Agent: Mechanistic Insights and Metabolic Regulation

**DOI:** 10.3390/ijms27125609

**Published:** 2026-06-22

**Authors:** Sedef Ziyanok-Demirtas, Irem Serin

**Affiliations:** Department of Biology, Faculty of Science and Arts, Bursa Uludag University, Bursa 16059, Turkey

**Keywords:** *G. sylvestre*, diabetes mellitus, gymnemic acids, phytochemicals, molecular mechanisms, diabetic complications, phytotherapy

## Abstract

Diabetes mellitus (DM) is a complex metabolic disorder characterized by chronic hyperglycemia and represents a major global public health concern due to its rapidly increasing prevalence. Although current pharmacological therapies effectively achieve glycemic control, their long-term use is limited by adverse effects, high costs, patient compliance issues, and increasing interest in safer, multi-targeted therapeutic strategies. In this context, plant-derived bioactive compounds have gained attention as complementary or alternative approaches to metabolic disease management. *Gymnema sylvestre* (Retz.) R.Br. ex Sm (GS), traditionally known as “gurmar” (“sugar destroyer”), is one of the most extensively studied medicinal plants with significant antidiabetic potential. This review evaluates the antidiabetic effects of *G. sylvestre*, focusing on its phytochemical composition, molecular mechanisms, and impact on diabetes-related complications. Major bioactive constituents, including triterpenoid saponins (gymnemic acids), gurmarin-like peptides, flavonoids, and sterols, regulate glucose homeostasis, inhibit intestinal glucose absorption, preserve pancreatic β-cell function, stimulate insulin secretion, modulate lipid metabolism, and suppress inflammatory signaling pathways. Experimental and clinical evidence indicates that *G. sylvestre* modulates oxidative stress and inflammation associated with complications such as nephropathy, neuropathy, retinopathy, vascular dysfunction, and dyslipidemia. This review adopts a mechanism-oriented framework integrating phytochemical structure–molecular target–metabolic outcome relationships and discusses emerging strategies, including nanotechnology-based delivery systems, molecular docking, and multi-component phytotherapy. Overall, *G. sylvestre* represents a promising multi-target phytotherapeutic agent, highlighting directions for future mechanistic and clinical research.

## 1. Introduction

Diabetes mellitus (DM) is a complex, multifactorial metabolic disease characterized by chronic hyperglycemia resulting from impaired insulin secretion, insulin resistance, or both [1,2,3]. Due to its rapidly increasing prevalence influenced by a sedentary lifestyle and changes in dietary habits, it constitutes a significant global public health burden [4]. In addition to disturbances in glucose homeostasis, DM is associated with systemic metabolic disorders, oxidative stress, chronic inflammation, and the progressive development of microvascular and macrovascular complications, such as nephropathy, neuropathy, retinopathy, and cardiovascular diseases [5,6,7]. Under normal physiological conditions, glucose homeostasis is tightly regulated by insulin secreted from pancreatic β-cells, which maintains metabolic balance by enhancing glucose uptake in peripheral tissues [3]. However, chronic hyperglycemia activates multiple molecular pathways that disrupt the intracellular metabolic balance and cause tissue damage. These pathways include the polyol pathway, formation of advanced glycation end products (AGEs), activation of protein kinase C (PKC), hexosamine pathway, and activation of poly (ADP-ribose) polymerase (PARP), all of which are closely related to oxidative stress and inflammation [5]. These mechanisms play a central role in the development and progression of diabetes-related complications. Although insulin and oral hypoglycemic agents are effective in achieving glycemic control, their long-term use is associated with various limitations, such as side effects, diminished efficacy over time, high costs, and poor patient compliance [8]. These limitations have increased interest in alternative and complementary treatment approaches based on natural products that can act on multiple biological targets [3]. In this context, medicinal plants are important sources of bioactive compounds capable of modulating multiple metabolic pathways simultaneously. Among these, *Gymnema sylvestre* (Retz.) R.Br. ex Sm., a perennial woody climber belonging to the family Apocynaceae, has attracted significant scientific interest because of its antidiabetic potential [9,10]. Traditionally known as “gurmar” (“sugar destroyer”), this plant is widely used in Ayurvedic medicine for managing diabetes and related metabolic disorders [9,11]. The pharmacological effects of *G. sylvestre* are largely attributed to its rich phytochemical content, including triterpenoid saponins (gymnemic acids), peptide compounds such as gurmarin, flavonoids, and sterols [12,13]. These bioactive compounds exhibit antidiabetic effects via multiple mechanisms, such as the inhibition of intestinal glucose absorption [14,15], stimulation of insulin secretion and protection of pancreatic β-cells [16,17], regulation of lipid metabolism [18,19], suppression of oxidative stress and inflammation [20,21,22], and modulation of sweet taste perception at the receptor level [3,23]. Although the number of experimental and clinical studies examining the biological effects of *G. sylvestre* is increasing, the current literature tends to focus on a limited number of mechanisms. Comprehensive evaluations that holistically reveal the relationships between phytochemical composition, molecular targets, and metabolic outcomes remain limited. Therefore, this review aims to comprehensively and mechanistically evaluate the antidiabetic potential of *G. sylvestre* extracts. The relationships among the bioactive components of these plants, their molecular mechanisms of action, and their metabolic effects are discussed within an integrative framework. In addition, novel translational approaches, such as molecular docking analyses, nanotechnology-based delivery systems, and polyherbal formulations, are critically assessed in terms of future research and clinical applications.

## 2. Materials and Methods

This review was prepared through a comprehensive literature search aimed at identifying studies investigating the biological and pharmacological effects of *Gymnema sylvestre* on diabetes mellitus and related complications.

### 2.1. Literature Search Strategy

Relevant English-language studies published up to February 2026 were systematically searched using the PubMed, Scopus, and Web of Science databases. During the search, the keywords “*Gymnema sylvestre*”, “*G. sylvestre*,” “diabetes mellitus,” “gymnemic acids,” “phytochemicals,” “molecular mechanisms,” “diabetic complications,” and “phytotherapy” were used. Relevant studies were systematically searched using the PubMed, Scopus, and Web of Science databases. During the search, the keywords “*Gymnema sylvestre*”, “*G. sylvestre*,” “diabetes mellitus,” “gymnemic acids,” “phytochemicals,” “molecular mechanisms,” “diabetic complications” and “phytotherapy” were used.

### 2.2. Inclusion and Exclusion Criteria

Studies were included if they (i) focused on *G. sylvestre,* (ii) reported antidiabetic or metabolic effects, and (iii) used in vitro, in vivo, or clinical models or were review articles. Only publications in English were included. Studies lacking sufficient methodological information, not directly related to diabetes, or containing repetitive data were excluded from the review.

### 2.3. Data Collection and Evaluation

The study design, model type, extract/compound used, dose, and biological effects were compiled and qualitatively evaluated.

### 2.4. Methodological Evaluation and Limitations

Considering the heterogeneity in the literature, experimental design, extract standardization, and dose differences were assessed. Differences among in vitro, in vivo, and clinical studies were addressed from a translational perspective, emphasizing that factors such as bioavailability, metabolic conversion, and study duration may limit clinical translation.

## 3. Botanical Characteristics, Geographical Distribution and Traditional Uses

### 3.1. Botanical Characteristics

*G. sylvestre* is a perennial, major climbing plant. Its stem is cylindrical and typically covered with fine hairs, whereas the root system generally develops as a taproot. The leaves are oppositely arranged, simple, and elliptic to ovate, measuring approximately 3–5 cm in length and 1–3 cm in width.

**Figure 1:**
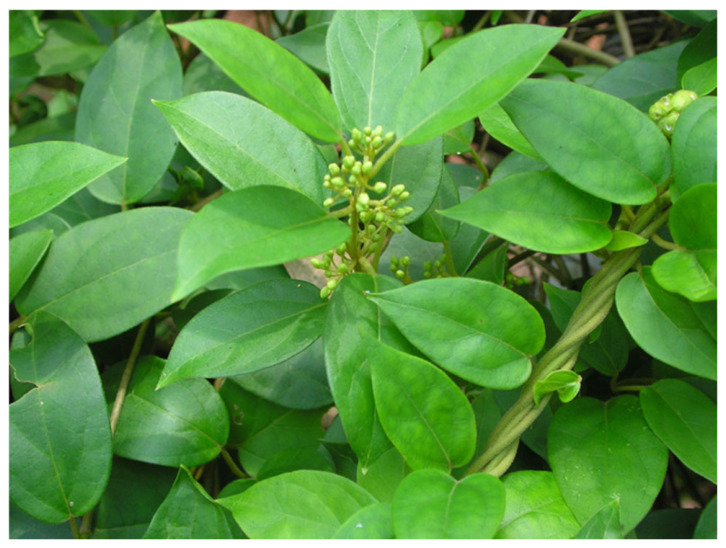
Representative photograph of *Gymnema sylvestre* (Licensed under CC BY 2.0).

The flowers are small, yellow, and arranged in umbellate cymose inflorescences (Figure 1). The fruits develop as lanceolate or cylindrical follicles, and the seeds are thin, winged, and elliptic, measuring approximately 1–3 cm in length. The flowering period typically begins in August and continues until March [9,24].

### 3.2. Geographical Distribution and Traditional Use of Gymnema sylvestre

**Figure 2:**
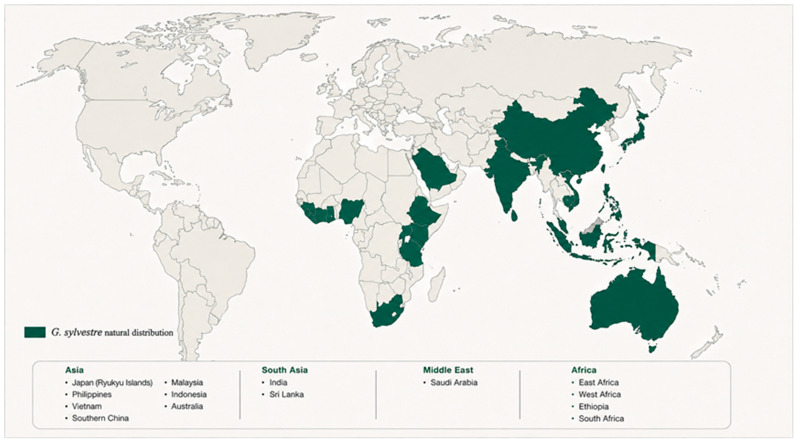
Geographical distribution map of Gymnema sylvestre. This figure was generated using the DALL-E 3 image generation tool integrated into ChatGPT (OpenAI) [https://chat.openai.com/, accessed on 18 May 2026].

*G. sylvestre* is a wild medicinal plant with a wide natural distribution across tropical and subtropical regions. Its geographical range extends from Japan (Ryukyu Islands), the Philippines, Vietnam, China, Malaysia, Indonesia, and Australia in the east to India, Sri Lanka, Saudi Arabia, and several regions of the African continent (including East and West Africa, Ethiopia, and South Africa) in the west [10,25]. The geographical distribution and traditional uses of *G. sylvestre* are presented in an integrated schematic format (Figure 2).

*G. sylvestre* has been used for centuries in traditional medicine systems, particularly in India, as well as in regions such as Japan, Vietnam, and Australia. Due to its ability to suppress sweet taste perception, the plant is commonly known as “gurmar,” meaning “sugar destroyer” in Hindi. It is widely recognized as a natural therapeutic agent for managing diabetes mellitus and other metabolic disorders [9,10]. This plant, which grows in different regions of the world, is known by various local names, including Chigengteng (China) and Gurmar (India) [10].

The pharmacological properties of *G. Sylvestre* have attracted scientific interest since early periods, with the first experimental studies on its antidiabetic effects dating back to the 1930s [11]. In classical Ayurvedic texts, including the *Charaka Samhita* and *Sushruta Samhita*, this plant is described in detail and identified as a therapeutic agent major management of metabolic diseases [9].

In Ayurvedic practice, *G. Sylvestre* is a key component of several traditional formulations, such as Mahavisagarbha Taila, Ayaskrti, Nyagrodhadi Churna, and Mrtasanji Vani Sura. Different plant parts, including leaves, roots, flowers, fruits, and bark, are utilized for various therapeutic purposes, depending on the disease and mode of application. Its traditional uses extend beyond metabolic and endocrine disorders to include gastrointestinal, hepatic, respiratory, cardiovascular, dermatological, and ophthalmic conditions [9,11,26].

## 4. Bioactive Constituents of *Gymnema sylvestre*

**Figure 3:**
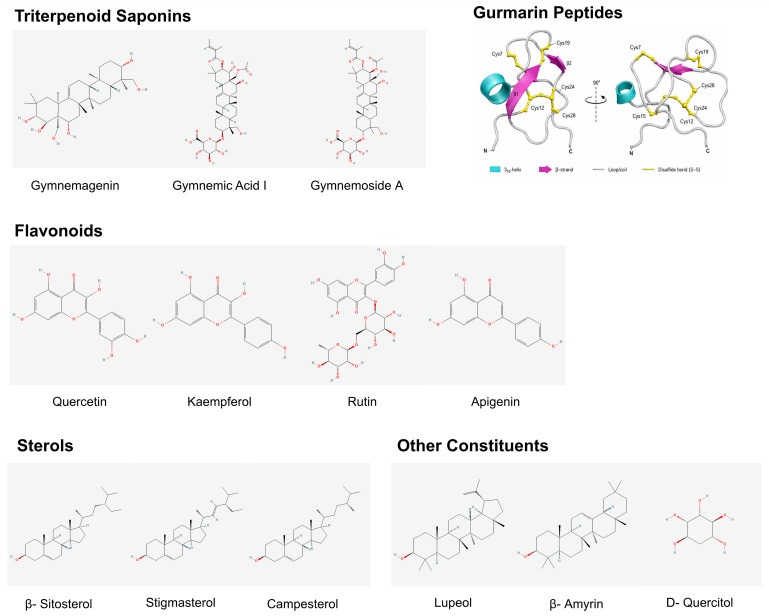
Major phytochemical compounds identified in *Gymnema sylvestre* (PubChem-based structure).

*G. sylvestre* is a medicinal plant characterized by a rich diversity of secondary metabolites that are heterogeneously distributed across different plant parts. Phytochemical analyses conducted by Devi et al. and Shah et al. demonstrated that the plant possesses a complex chemical profile rich in triterpenoid saponins, flavonoids, tannins, alkaloids, steroids, and various organic acids. Among these compound classes, the saponin fraction is largely responsible major antidiabetic and hypocholesterolemic effects, while terpenoids contribute to the regulation of glucose metabolism, and steroidal compounds exhibit various biological effects on the central nervous system [12,13]. The major bioactive constituents of *G. sylvestre* are shown in Figure 3.

The phytochemical composition of *G. sylvestre* varies significantly depending on the morphological part of the plant. Various bioactive compounds, including alkaloids, flavones, anthraquinones, hentriacontane, pentatriacontane, α- and β-chlorophylls, phytin, resins, and D-quercitol, have been identified in the flower, leaf, and fruit tissues [11]. In addition, it has been reported that proline is not present in significant amounts in *G. sylvestre* leaves under normal physiological conditions; however, its accumulation increases markedly under biotic and abiotic stress conditions [25].

The antidiabetic activity of this plant is primarily attributed to its leaf tissue. Indeed, the highest concentration of gymnemic acids has been reported in the shoot tips (54.29 mg/g dry weight), whereas the lowest concentration is found in the seeds (1.31 mg/g dry weight) [25]. Furthermore, the structure and composition of phytochemical constituents vary among different plant parts, with compounds present in the stem differing significantly from those in the leaves [27]. The main bioactive compounds identified in *G. sylvestre* are illustrated in Figure 3.

The primary class of compounds responsible major antidiabetic effects of *G. sylvestre* is believed to be gymnemic acids. These triterpenoid saponins inhibit intestinal glucose absorption, support pancreatic β-cell function, and increase insulin secretion. Triterpenoid saponins are the most characteristic and therapeutically important bioactive compounds found in *G. sylvestre*. It has been reported that a significant portion of GS leaves consists of triterpenoid saponins, particularly derivatives, and that these compounds are mainly responsible major plant’s fundamental pharmacological activities [28]. Di Fabio et al. examined the isolation, chemistry, and bioactivities of triterpenoid compounds oxidized at the C-23 position and summarized the pharmacological properties of these saponins according to their mechanisms as follows [29]: Suppression of Sweetness Perception (Antisweet Effect): Gymnemic acids (GA) forms I–VI, VIII–XII, and XV–XVIII can completely suppress the sensation of sweetness from sucrose. This effect is directly proportional to the number of acyl groups in the molecule, whereas GA VII, prosapogenin, and gymnemagenin do not exhibit this activity. Insulin secretion and antihyperglycemic activity: Gymnemic acids IV is a multifunctional antihyperglycemic agent owing to its sweetness-suppressing, glucose uptake-inhibiting, and intestinal glucosidase-inhibiting properties. Furthermore, gymnemosaponin V and gymnemic acids I, II, III, and IV have been shown to stimulate insulin secretion from the pancreas and increase insulin levels in the blood plasma of diabetic models. Inhibition of Intestinal Glucose Absorption: GA II, III, and V have been reported to strongly inhibit glucose absorption in the small intestine, and GA I, VII, and certain gymnemocides (a and b) also contribute to this process. These compounds are thought to support the repair of β-cells by increasing gastric inhibitory polypeptide (GIP) secretion [29]. In addition to leaves, studies on stem extracts have identified different triterpenoid structures. Liu et al. [27] isolated conduritol A, stigmasterol, lupeol, and a new triterpenoid saponin from the stem, reporting that these compounds have the ability to inhibit non-enzymatic glycation of proteins under in vitro conditions. Additionally, Shah et al. isolated four more new triterpenoid saponins from this plant, named Gymnemasin A, B, C, and D [13]. In addition to triterpenoid saponins, *G. sylvestre* contains peptide-based compounds that inhibit sweet taste receptor activity. The best-characterized of these is gurmarin, a cysteine-rich polypeptide composed of approximately 35 amino acids. Gurmarin contains three disulfide bridges and exhibits a compact protein folding motif known as a “cystine-knot,” which provides high stability against proteolytic enzymes, temperature changes, and chemical degradation. Recent transcriptomic analyses have revealed that *G. sylvestre* contains not only classical gurmarin (Gur-1) but also numerous gurmarin-like peptides belonging to the same peptide family. These peptides, numbered Gur-2 through Gur-9, have been shown to interact with the sweet taste receptor heterodimer T1R2/T1R3. Molecular modeling studies have demonstrated that Gur-1 and Gur-2 bind to the cysteine-rich domain (CRD) and transmembrane domain (TMD) of the T1R2 subunit. This interaction may contribute to the suppression of sweet taste perception and, consequently, to a reduction in sugar intake [23]. Overall, the antidiabetic effects of *G. sylvestre* have been largely attributed to the presence of triterpenoid saponins. However, other compounds, including flavonoids, sterols, and peptide-based molecules such as gurmarin, may also contribute to the plant’s overall therapeutic effects through complementary mechanisms involving antioxidant activity, metabolic regulation, and taste modulation. Therefore, the pharmacological activity of GS arises not from a single molecule but from synergistic interactions among multiple bioactive compounds.

The major phytochemical groups identified in *G. sylvestre* and their diabetes-related biological activities are summarized in Table 1.

The phytochemical constituents of *G. sylvestre* exhibit diverse biological activities, including antioxidant, anti-inflammatory, hypoglycemic, and lipid-regulating effects. In particular, gymnemic acids play an important role in the regulation of glucose metabolism, and flavonoids and other phenolic compounds contribute to the suppression of oxidative stress and inflammation. These biological effects support the therapeutic potential of *G. sylvestre* in treating diabetes and related metabolic disorders.

In addition to triterpenoid saponins, *G. sylvestre* leaves contain various minor constituents and nutritional components. Gas chromatography-mass spectrometry (GC–MS) analyses performed using different solvents have demonstrated that the plant contains more than 38 phytocompounds with natural antioxidant potentials. Among these, biologically active molecules such as squalene, phytol, n-hexadecanoic acid, and stigmasterol have been consistently identified across different extracts [31].

The plant also contains various sterols, flavonoids, and glycosides. Sterols, such as stigmasterol, lupeol, and β-amyrin, are known for their hypoglycemic and anticancer properties and may enhance the effects of saponins [8]. Among flavonoid compounds, kaempferol derivatives and glycosides, such as Alternoside II, have been reported. These flavonoids exhibit antioxidant and anti-inflammatory properties, which may play a significant role in preventing diabetic complications.

*G. sylvestre* is also rich in mineral elements. It has been reported that the leaf matrix consists of approximately 22% cellulose, 7.3% calcium oxalate, 5.5% organic acids, and 4.8% lignin [12]. In addition, the plant contains significant amounts of inorganic elements, such as calcium (Ca), potassium (K), magnesium (Mg), iron (Fe), and zinc (Zn). These elements can contribute to metabolic regulatory mechanisms, particularly by acting as cofactors in insulin synthesis and secretion [32].

Various analytical methods have been developed for the isolation and quantitative analysis of gymnemic acids derivatives in *G. sylvestre* [25]. Extraction procedures are generally performed using methanol-based methods that provide high yields. It has been reported that extraction with 90% methanol using the Hooper method yields the maximum amount of gymnemic acids from the leaves. In addition, ultrasound-assisted extraction methods can reduce extraction time while minimizing the thermal degradation of target analytes.

Gravimetric methods can be used to estimate the total gymnemic acids content. High-performance liquid chromatography (HPLC) is widely employed for precise analyses. In this method, gymnemic acids are typically not measured directly but quantified via their aglycone form, gymnemagenin. The detection limit of gymnemagenin has been reported to be approximately 1 µg/mL [25]. Furthermore, validated HPLC methods based on the quantitative analysis of deacylated gymnemic acids are available.

High-performance thin-layer chromatography (HPTLC) is commonly used for chemical fingerprinting and standardization of *G. sylvestre* extracts. Gymnemagenin is typically used as a reference compound in this technique [13].

However, despite the availability of various extraction and analytical techniques, some methodological limitations must be considered when using these techniques. The yield and composition of gymnemic acids can vary significantly depending on the extraction solvent, method, plant part, and geographical origin of raw material. In particular, methanol-based extraction methods, although effective, may not fully reflect the composition of conventionally used preparations. Furthermore, the indirect quantification of gymnemic acids via gymnemagenin can lead to variability and affect analytical accuracy. Although HPLC methods are commonly used, differences in calibration strategies, reference standards, and validation parameters can lead to inconsistencies among studies. Therefore, the development of standardized, validated, and reproducible analytical protocols is crucial for ensuring the comparability and reliability of the results. Moreover, the doses used in experimental studies are often considerably higher than those achievable through dietary intake, which may raise concerns regarding their direct translational applicability to clinical settings.

## 5. Molecular Mechanisms Underlying the Antidiabetic Effects of *Gymnema sylvestre*

The antidiabetic activity of *G. sylvestre* (GS) is mediated through a complex network of complementary molecular mechanisms, including stimulation of insulin secretion, inhibition of intestinal glucose absorption, modulation of insulin signaling pathways, suppression of sweet taste perception, and regulation of inflammatory processes [33,34]. In addition to conventional in vivo and in vitro findings, recent in silico and network-based approaches have provided deeper insights into the multi-target molecular interactions of GS bioactive compounds.

### 5.1. Pancreatic Beta Cell Dysfunction and Restoration

Diabetogenic agents, such as streptozotocin (STZ) and alloxan, which exhibit selective cytotoxicity toward pancreatic β-cells, are widely used to establish experimental diabetes models. These agents induce β-cell damage primarily through excessive generation of reactive oxygen species (ROS), oxidative stress, and DNA damage, ultimately leading to impaired insulin secretion and the development of hyperglycemia.

The antidiabetic effects of *G. sylvestre* have been recognized since the early 20th century, with studies dating back to the 1930s reporting reductions in urinary glucose levels in diabetic individuals. In experimental models, GS extracts have consistently shown to improve glucose homeostasis and support pancreatic β-cell regeneration [16].

In STZ- and alloxan-induced diabetic animal models, GS administration significantly increased serum insulin levels and reduced glycated hemoglobin (HbA1c), indicating improved glycemic control. Mechanistically, these effects have been associated with enhanced glucose utilization through the regulation of key metabolic enzymes, including phosphorylase, gluconeogenic enzymes, and sorbitol dehydrogenase [17].

Long-term administration of GS (20–60 days; approximately 20 mg/day) in STZ-diabetic rats has been reported to normalize blood glucose levels, while dose-dependent hypoglycemic effects have been observed across a wide range of doses (50–500 mg/kg) [35,36].

In addition, GS treatment reduces oxidative stress in pancreatic tissue, as evidenced by decreased malondialdehyde (MDA) levels and increased activities of antioxidant enzymes, such as superoxide dismutase (SOD) and catalase (CAT). Histopathological analyses further revealed that degeneration of the islets of Langerhans and β-cell loss observed in the diabetic control groups were markedly attenuated following GS treatment [8,14].

**Figure 4:**
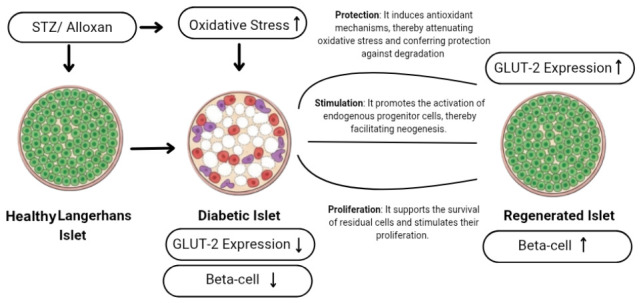
Schematic representation of pancreatic beta cell damage in experimental diabetes models and the regenerative repair mechanisms triggered by *G. sylvestre* treatment. Arrows indicate the direction of the proposed pathological and regenerative processes within pancreatic islets.

Collectively, these findings indicate that *G. sylvestre* exerts antidiabetic effects through multiple complementary mechanisms, including enhancement of insulin secretion, protection of pancreatic β-cells against oxidative and inflammatory damage, and regulation of glucose metabolism. These processes are schematically illustrated in Figure 4.

In STZ-induced diabetic rat models, pronounced histopathological alterations are observed in the pancreatic tissue. These include severe disruption of islet architecture, islet atrophy, marked reduction in β-cell population, and cytoplasmic degranulation.

In parallel with these morphological changes, the expression of glucose transporter type 2 (GLUT2), which plays a critical role in glucose uptake by β-cells, has been shown to decrease to negligible levels. This dramatic reduction in GLUT2 expression leads to impaired pancreatic β-cell function and severe suppression of insulin secretion [37,38].

Similar histopathological findings have also been observed in alloxan-induced diabetes models. Alloxan induces excessive ROS production in pancreatic β-cells, leading to selective necrosis and significant cellular loss in the islets of Langerhans. This process results in a rapid decline in insulin production capacity and the development of diabetic metabolic disturbances [39].

The effects of *G. sylvestre* (GS) treatment on pancreatic tissue extend beyond glycemic control and encompass regenerative processes that restore the structural and functional integrity of pancreatic β-cells. In experimental diabetic models, GS-treated groups showed significant improvements in islet architecture, an increased β-cell population, and enlargement of islet diameter. These findings suggest that GS can partially reverse structural damage in pancreatic tissue [38].

At the molecular level, GS treatment has been shown to modulate transcription factors critical for pancreatic development and insulin synthesis. Muzaffar et al. [40] showed that *G. sylvestre* (GS) leaves increased plasma insulin levels and stimulated insulin gene transcription in hyperglycemic rats. It was suggested that the initial increase in plasma insulin levels was due to incretins triggering insulin secretion by increasing β-cell membrane permeability, and that this effect may be related to increased secretion rather than insulin synthesis. However, incretins have been reported to play a role in the transcriptional activation of Pdx-1 (Pancreatic and Duodenal Homeobox-1), a transcription factor critical for pancreatic development and insulin synthesis, and that Pdx-1 expression is increased in conjunction with insulin, which in turn enhances insulin synthesis by increasing Ins-1 gene expression. At the molecular level, it has been shown that GS treatment modulates genes associated with pancreatic development and insulin biosynthesis and reduces necrotic and degenerative changes in diabetic groups. These findings suggest that GS has a key molecular mechanism of action that supports β-cell function and increases insulin production [40].

These findings suggest that GS stimulates β-cell function and may activate genetic pathways involved in cellular proliferation and β-cell neogenesis [41,42].

Another important mechanism underlying β-cell protection is the preservation of cellular energy metabolism. Akram et al. reported that under conditions of high oxidative stress, excessive activation of the nuclear enzyme poly (ADP-ribose) polymerase (PARP) leads to depletion of NAD^+^ and ATP stores in β-cells, triggering necrotic cell death. GS reduces oxidative stress levels, limits PARP activation, and thereby preserves cellular energy balance, supporting β-cell viability because of its strong antioxidant capacity [5].

The protective effects of GS on pancreatic β-cells have also been confirmed in in vitro cell culture studies. Al-Romaiyan et al. investigated the effects of GS extracts using three-dimensional pseudoislet models derived from human β-cell lines (1.1B4). The results showed that, in particular, the ethanolic extract significantly reduced apoptosis in β-cells exposed to high glucotoxicity and contributed to the preservation of islet morphology. The authors suggested that this effect may be associated with the restoration of islet structure and preservation of endogenous insulin reserves [43].

Furthermore, GS treatment has been reported to induce the expression of GLUT2 transporter proteins in the pancreatic β-cell membrane, which plays a critical role in glucose transport [38]. Restoration of GLUT2 expression is essential for re-establishing glucose sensing capacity and regulating insulin secretion.

Overall, these findings indicate that *G. sylvestre* exerts a multi-target mechanism of action that supports pancreatic β-cell function and promotes both cellular regeneration and molecular recovery.

### 5.2. Insulin Signaling

*G. sylvestre* (GS) extracts, particularly the Om Santal Adivasi (OSA) fraction, have been shown to exert a direct insulin secretagogue effect on pancreatic β-cells. This effect is primarily mediated through the modulation of intracellular signaling pathways, including calcium-dependent mechanisms that facilitate insulin exocytosis. Experimental studies have demonstrated that GS bioactive compounds enhance glucose-stimulated insulin secretion (GSIS) while preserving β-cell viability under metabolic stress conditions, such as glucotoxicity and lipotoxicity [43,44]. In addition, both in vivo and in vitro evidence support the direct stimulatory effects of high-molecular-weight GS extracts on human pancreatic islets. Clinical findings have demonstrated that administration of a standardized OSA extract (1 g/day for 60 days) significantly reduced fasting and postprandial blood glucose levels while increasing serum insulin and C-peptide concentrations in patients with type 2 DM. Complementary in vitro analyses using isolated human islets further confirmed that OSA enhances insulin secretion even at low glucose concentrations and potentiates glucose-stimulated insulin release without compromising cell membrane integrity. These findings indicate that GS extracts not only stimulate insulin secretion but also preserve β-cell functionality and safety, supporting their potential as targeted insulinotropic agents [45].

Furthermore, GS extracts contribute to the maintenance of β-cell integrity and functional responsiveness, indicating a dual role in both insulin secretion and β-cell protection.

One of the primary mechanisms underlying the insulinotropic effect of GS involves the modulation of ion channels in the pancreatic β-cell membrane. Calcium imaging and electrophysiological studies have shown that GS bioactive compounds inhibit ATP-sensitive potassium (KATP) channels, thereby reducing potassium efflux from the cell. This inhibition induces membrane depolarization, which subsequently triggers the opening of voltage-dependent calcium channels (VDCC). The resulting increase in intracellular calcium influx plays a critical role in initiating insulin secretion.

**Figure 5:**
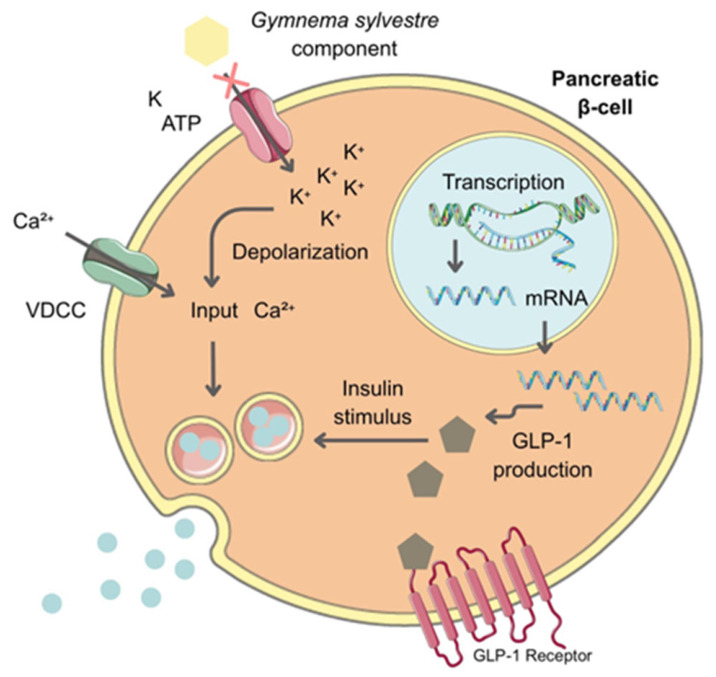
Schematic representation of electrophysiological and nuclear signaling pathways mediating the insulin-secretagogue effect of *G. sylvestre*. X: Inhibition of the ATP-sensitive potassium (KATP) channel. This figure was created using graphical elements from Servier Medical Art (https://smart.servier.com) under the CC BY 4.0 license [accessed on 24 May 2026].

Previous studies summarized by Jamadagni et al. demonstrated that GS extracts not only increase intracellular calcium levels but also activate protein kinase-mediated signaling pathways, thereby modulating insulin secretion through physiological mechanisms [25]. The elevation in intracellular calcium concentration, together with the activation of enzymatic signaling pathways, facilitates the fusion of insulin-containing secretory vesicles with the plasma membrane, leading to insulin release via exocytosis into the systemic circulation, as schematically illustrated in Figure 5.

This mechanism is comparable to that of sulfonylurea-class antidiabetic drugs that are commonly used in clinical practice [39]. However, GS-derived bioactive compounds, particularly gymnemic acids, have been reported to enhance β-cell glucose sensitivity and modulate insulin secretion in a glucose-dependent manner [43,44,46].

The effects of GS saponins extend beyond the stimulation of insulin secretion and involve the restoration of disrupted intracellular signaling networks under diabetic conditions. Akram et al. reported that this process is closely associated with the reactivation of the phosphatidylinositol-3-kinase/protein kinase B (PI3K/Akt) signaling pathway [5].

Under diabetic conditions, increased oxidative stress inhibits the PI3K/Akt pathway, leading to mitochondrial dysfunction and the initiation of cellular apoptosis. GS saponins have been shown to reactivate this pathway, thereby enhancing β-cell survival and stabilizing cellular metabolism. In addition to its metabolic role, insulin functions as a neurotrophic factor in maintaining neuronal function. Increased insulin secretion stimulated by GS may indirectly enhance neurotrophic support mechanisms, thereby exerting protective effects against complications, such as diabetic neuropathy [5].

The effects of GS on insulin secretion are not limited to ion channel modulation but are also mediated through the incretin system and transcriptional regulatory mechanisms.

Hydroalcoholic extracts of *G. sylvestre* (HAGS) increase proglucagon mRNA expression in pancreatic and enteroendocrine cells. The processing of proglucagon leads to the formation of glucagon-like peptide-1 (GLP-1), which binds to β-cell surface receptors and enhances glucose-dependent insulin secretion. These findings suggest that *G. sylvestre* supports cellular signaling mechanisms that regulate β-cell function and insulin secretion [22].

Incretin hormone secretion is closely associated with gut microbiota composition. GS administration has been reported to modulate intestinal microbiota by reducing the Firmicutes/Bacteroidetes ratio and increasing the abundance of short-chain fatty acid (SCFA)-producing bacteria, such as *Bacteroides* and *Lactobacillus*.

SCFAs, such as butyrate and acetate, produced as a result of these microbiota changes, not only support intestinal barrier integrity but also stimulate GLP-1 secretion from enteroendocrine cells, thereby enhancing insulin secretion via the incretin axis [19].

These findings indicate that *G. sylvestre* is a phytotherapeutic agent that modulates insulin signaling through multilayered mechanisms. Plant extracts enhance β-cell function and support glucose-dependent insulin secretion via ion channel modulation, restoration of intracellular signaling pathways, activation of the incretin axis, and regulation of gut microbiota.

### 5.3. Intestinal Glucose Absorption and Enzyme Inhibition

GS exerts significant antihyperglycemic effects by targeting the mechanisms of intestinal carbohydrate digestion and glucose absorption. The inhibition of α-amylase and α-glucosidase enzymes plays a central role in reducing postprandial hyperglycemia by delaying carbohydrate hydrolysis and glucose formation.

Both in vitro and in silico studies have demonstrated that GS-derived saponins, particularly gymnemasaponin V, exhibit a strong binding affinity for the catalytic sites of α-amylase and α-glucosidase. Molecular docking analyses revealed that gymnemasaponin V interacts with α-amylase with a binding energy of −9.7 kcal/mol, which is stronger than that of acarbose (−7.4 kcal/mol), suggesting potent enzyme inhibition. Similarly, gymnemasin B, gymnemasin C, and gymnemozide C showed high binding affinities toward α-amylase, whereas gymnemasaponin IV and gymnemasin B were identified as promising α-glucosidase inhibitors [47]. These interactions limit substrate access to the active site, thereby reducing carbohydrate digestion and glucose release into circulation [48].

In addition to enzyme inhibition, gymnemic acids structurally resemble glucose molecules and competitively interact with intestinal glucose receptors, thereby reducing glucose absorption [15]. GS compounds also inhibit sodium-dependent glucose transporter 1 (SGLT1), directly suppressing glucose transport into enterocytes [15,25]. These findings have been validated in experimental systems, including the Xenopus laevis oocyte model.

Furthermore, GS modulates intestinal physiology by altering the membrane potential and transporter activity, thereby limiting passive glucose diffusion. It also influences the gut microbiota composition by increasing the abundance of SCFA-producing bacteria and reducing that of pathogenic species, thereby improving intestinal integrity and metabolic homeostasis [19].

Collectively, GS regulates postprandial glycemia through coordinated mechanisms involving enzyme inhibition, receptor competition, transporter modulation, and microbiota regulation.

### 5.4. Insulin Sensitivity Enhancement and Peripheral Glucose Uptake

In the pathophysiology of type 2 DM, a reduction in the number of insulin receptors or impairment of receptor function is considered a key determinant in the development of insulin resistance. Decreased insulin receptor density weakens the responsiveness of target tissues to insulin, thereby limiting glucose transport into cells [22].

Although the insulin-sensitizing effects of *G. sylvestre* (GS) have been demonstrated in both preclinical studies and in diabetic patients, clinical responses may vary depending on the baseline glycemic status. A clinical study conducted in individuals with metabolic syndrome (MetS) reported that GS administration did not significantly alter insulin secretion or sensitivity. However, this outcome was attributed to the fact that the majority of participants (92%) exhibited normal glucose tolerance. Notably, although the placebo group showed decreased insulin sensitivity and compensatory hyperinsulinemia, these deteriorations were not observed in the GS-treated group, suggesting that GS may help stabilize metabolic function and prevent further metabolic decline [49].

Studies have demonstrated that hydroalcoholic extracts of *G. sylvestre* significantly upregulate insulin receptor gene expression in both pancreatic and peripheral tissues. This upregulation enhances insulin sensitivity and facilitates glucose uptake by improving receptor-mediated signaling efficiency [22].

Methanolic extracts of *G. sylvestre* have been shown to improve insulin sensitivity by enhancing glucose uptake, particularly in the skeletal muscle and adipose tissue, which are the primary sites of insulin-mediated glucose disposal [38,50].

Glucose transporter type 4 (GLUT4) plays a central role in insulin-dependent glucose uptake, and impaired GLUT4 translocation is a key factor in the development of insulin resistance. GS-derived bioactive compounds have been reported to increase GLUT4 expression and promote its translocation to the plasma membrane, thereby facilitating glucose entry into peripheral tissues and improving glycemic control [22,37,38].

In vitro studies using L6 skeletal muscle cells further demonstrated that GS extracts enhance glucose uptake by upregulating GLUT4 expression. Notably, the complete inhibition of this effect in the presence of cycloheximide (CHX), a protein synthesis inhibitor, indicates that this mechanism is dependent on de novo protein synthesis [51].

Clinical findings remained variable. A study conducted in individuals with metabolic syndrome reported that GS supplementation did not significantly alter insulin sensitivity; however, this was attributed to the normal baseline glucose tolerance of most participants. Importantly, the deterioration observed in the placebo group was not evident in the GS-treated group, suggesting a stabilizing effect on metabolic function [49].

Collectively, these findings suggest that *G. sylvestre* enhances peripheral glucose utilization primarily through GLUT4-mediated mechanisms, thereby improving insulin sensitivity.

The beneficial effects of *G. sylvestre* on insulin sensitivity are also mediated through the modulation of intracellular signaling pathways. Under hyperglycemic conditions, increased de novo diacylglycerol (DAG) synthesis leads to excessive activation of protein kinase C (PKC) isoforms, which impairs insulin signaling by disrupting insulin receptor substrate (IRS) phosphorylation. GS-derived compounds have been reported to modulate PKC activity, thereby contributing to the restoration of insulin signaling [5].

In addition, GS treatment has been shown to upregulates peroxisome proliferator-activated receptor gamma (PPAR-γ), a nuclear transcription factor that plays a critical role in regulating glucose and lipid metabolism. Activation of PPAR-γ enhances insulin sensitivity and promotes glucose uptake in peripheral tissues [22,37].

GS may influence adiponectin-mediated pathways, which are key regulators of metabolic homeostasis. Clinical evidence suggests that GS administration may improve insulin sensitivity, potentially through adiponectin-related mechanisms [49], whereas in vitro studies have demonstrated enhanced glucose uptake and modulation of key metabolic regulators, including AMP-activated protein kinase (AMPK) and peroxisome proliferator-activated receptor alpha (PPAR-α) signaling pathways [51].

In summary, these findings indicate that *G. sylvestre* improves insulin sensitivity by coordinating the regulation of intracellular signaling networks involved in glucose and lipid metabolism.

Dysbiosis contributes to insulin resistance through chronic low-grade inflammation and metabolic endotoxemia. *G. sylvestre* (GS) treatment has been shown to restore gut microbiota balance, increase the abundance of short-chain fatty acid (SCFA)-producing bacteria, strengthen intestinal barrier integrity, and reduce lipopolysaccharide (LPS)-induced inflammation, thereby improving insulin signaling [19].

These microbiota-mediated effects are closely associated with reduced systemic inflammation and improved metabolic homeostasis. By modulating gut microbial composition and enhancing intestinal barrier function, GS may limit endotoxin translocation into the circulation and attenuate inflammation-driven insulin resistance.

Collectively, these findings indicate that *G. sylvestre* improves insulin sensitivity indirectly by regulating gut microbiota and inflammation-related pathways.

In addition to these mechanisms, in silico analyses have demonstrated that GS-derived compounds interact with key metabolic enzymes involved in lipid metabolism, including HMG-CoA reductase, fatty acid synthase (FAS), and pancreatic lipase. Notably, gymnemasaponin IV and gymnemic acid I tigloyl exhibited strong binding affinities for HMG-CoA reductase, suggesting the potential for inhibition of cholesterol biosynthesis. These findings support the role of GS in improving lipid metabolism and insulin sensitivity through multi-targeted enzymatic regulation [47,52].

### 5.5. Sweet Taste Receptor Modulation (Anti-Sweet Activity)

**Figure 6:**
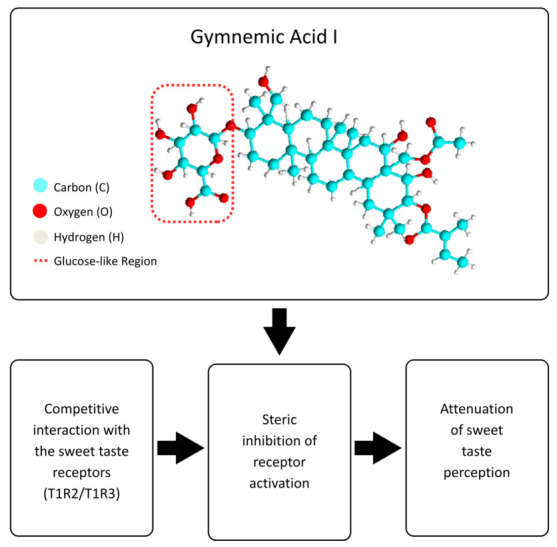
Proposed mechanism of sweet taste suppression by gymnemic acids. The structure of Gymnemic Acid I was reconstructed using structural information obtained from PubChem and redrawn using ChemDraw Professional 2025 (v2025.2.5).

The anti-sweet activity of GS is mediated through competitive interactions between gymnemic acids and peptide-based compounds with sweet taste receptors (T1R2/T1R3). These compounds sterically block receptor activation, thereby suppressing sweet taste perception (Figure 6) [10,15].

Advanced in silico analyses have further elucidated these interactions. Molecular docking and simulation studies have demonstrated that gurmarin-derived peptides, including Gur-1 and Gur-2 isoforms, exhibit a high binding affinity for the extracellular Venus Flytrap Domain (VFT) of T1R2/T1R3 receptors. These interactions stabilize the open conformation of the receptor, thereby preventing signal transduction. Notably, Gur-2 exhibits stronger structural stability and potential functional relevance in human receptors.

In addition, SAR studies have revealed that structural features, such as glucuronic acid moieties and acyl substitutions, significantly influence receptor binding affinity and inhibitory activity [23,29]. These findings support a molecular mimicry-based mechanism underlying sweet taste suppression and its contribution to dietary glucose regulation.

### 5.6. Anti-Inflammatory Mechanisms

The pathophysiology of type 2 diabetes is characterized by complex interactions between chronic low-grade inflammation, oxidative stress, and pancreatic β-cell dysfunction [1]. Hyperglycemia-induced increases in reactive oxygen species (ROS) lead to oxidative damage to cellular components, triggering inflammatory responses and contributing to metabolic dysregulation [53].

In this context, the anti-inflammatory effects of *G. sylvestre* (GS) extracts are closely associated with the modulation of key molecular signaling pathways and suppression of inflammation at both cellular and tissue levels. Chronic inflammation is a central process in the progression of diabetes, contributing to impaired pancreatic β-cell function and the development of insulin resistance in peripheral tissues. Experimental and clinical studies have demonstrated that GS extracts regulate inflammatory biomarkers, including reduced C-reactive protein (CRP) levels and suppression of pro-inflammatory cytokines, such as interleukin-6 (IL-6) and TNF-α [7,8].

At the molecular level, GS bioactive compounds suppress chronic low-grade inflammation (meta-inflammation) by targeting multiple signaling pathways involved in the inflammatory processes. A key mechanism involves the inhibition of nuclear factor kappa B (NF-κB) activation, limiting its translocation to the nucleus, thereby reducing the expression of pro-inflammatory cytokines, including TNF-α, IL-1β, and IL-6 [39].

Under hyperglycemic conditions, the interaction between advanced glycation end products (AGEs) and their receptor (RAGE) is a major trigger of NF-κB-mediated inflammatory signaling. GS extract has been reported to modulate the AGE–RAGE axis, weakening this interaction and consequently reducing cytokine production [5]. In addition, hyperglycemia-induced metabolic flux through the hexosamine biosynthetic pathway alters transcription factors, such as Sp-1, leading to increased inflammatory gene expression. GS compounds are suggested to regulate glucose metabolism, thereby limiting this shift and indirectly suppressing inflammatory signaling [5].

These molecular findings were supported by broader mechanistic and systems-level analyses. Network pharmacology studies have demonstrated that GS bioactive compounds interact with multiple inflammation-related targets, including TNF, IL1B, and PPARG, which are central regulators of metabolic inflammation and insulin resistance. Pathway enrichment analyses further highlighted the involvement of AGE–RAGE signaling, TNF-mediated pathways, and inflammatory cascades, supporting the multi-target anti-inflammatory potential of the plant.

Collectively, these mechanisms contribute to the preservation of pancreatic β-cell integrity, reduction in cytokine-induced cellular damage, and attenuation of inflammation-associated insulin resistance at both the molecular and systemic levels.

Chronic inflammation also plays a critical role in the development of complications associated with diabetes. In particular, prolonged inflammatory phases and increased matrix metalloproteinase-9 (MMP-9) activity contribute to impaired wound healing in diabetes. Daisy et al. demonstrated that GS- and curcumin-loaded nanocomposite biomaterials reducedMMP-9 activity, limited collagen degradation, and promoted tissue regeneration in diabetic wounds [54].

Furthermore, GS extract has been reported to modulate macrophage infiltration, accelerating the transition from the inflammatory to proliferative phase of wound healing. These findings indicate that GS exerts not only anti-inflammatory but also tissue-repair effects in diabetes-related complications.

## 6. Integrated Therapeutic Evidence, Clinical Applications, and Translational Considerations of *Gymnema sylvestre*

This section provides a comprehensive overview of the therapeutic effects of *G. sylvestre* (GS), integrating experimental findings, clinical evidence, safety considerations, and translational challenges. The biological activity of GS has been investigated in vitro, in animal models, and in human studies, each contributing different levels of mechanistic and physiological insight.

To provide a structured overview of the available evidence, experimental and clinical studies evaluating the biological effects of GS are summarized in Table 2. While in vitro studies provide mechanistic insights, in vivo and clinical studies offer more physiologically relevant results. However, variations in extract composition, dosage, and study design necessitate careful interpretation of their translational relevance.

The studies summarized in Table 2 include a wide range of experimental models used to investigate the antidiabetic potential of *G. sylvestre*, including in vitro systems, animal models, and clinical trials. While in vitro studies provide mechanistic insights at the molecular level, in vivo and clinical studies offer more physiologically relevant findings than in vitro studies. However, variations in extract composition, dosage, treatment duration, and outcome measures among studies may limit their direct comparability. Therefore, careful consideration of the level of evidence and translational relevance is required when interpreting these findings. Overall, the available evidence highlights the multifaceted therapeutic potential of *G. sylvestre* in diabetes management.

### 6.1. Effects on Microvascular Complications

*G. sylvestre* (GS) exhibits significant protective effects against diabetes-associated microvascular complications, including nephropathy, neuropathy, and retinopathy. Under diabetic conditions, GS demonstrates strong renoprotective activity at both the biochemical and histopathological levels. Elevated serum urea and creatinine levels, which are major indicators of renal dysfunction, are significantly reduced following GS treatment, suggesting the restoration of glomerular filtration capacity [59,60]. Histological analyses further demonstrated the inhibition of Bowman’s capsule expansion and preservation of the glomerular architecture. GS also modulates vascular endothelial growth factor (VEGF) expression, thereby reducing vascular leakage and improving renal microangiopathy [50].

The neuroprotective effects of GS are closely associated with its antioxidant and anti-inflammatory activities. Diabetic neuropathy is primarily driven by oxidative stress, mitochondrial dysfunction and chronic inflammation. GS treatment reduces lipid peroxidation, as evidenced by decreased malondialdehyde (MDA) levels, while simultaneously enhancing antioxidant defense mechanisms by increasing superoxide dismutase (SOD), catalase (CAT), and glutathione peroxidase (GPx) activities [37]. Furthermore, GS suppresses pro-inflammatory cytokines, such as TNF-α and IL-1β, indicating the modulation of neuroinflammatory signaling pathways. Functional improvements have also been demonstrated by increased nociceptive thresholds in behavioral studies. Histopathological findings revealed reduced axonal degeneration and improved myelin sheath integrity in the treatment group. Additionally, GS restores nerve growth factor (NGF) and insulin-like growth factor (IGF) levels, thereby supporting neuronal repair and survival [37].

GS also exerts protective effects against diabetes-induced retinal injuries. Structural abnormalities, including choroidal thickening, vascular degeneration, and endothelial damage, are significantly attenuated following treatment [62]. Moreover, molecular docking analyses suggest that GS-derived triterpenoid saponins inhibit aldose reductase (ALR2), thereby reducing sorbitol accumulation and osmotic stress, which are critical contributors to cataract formation and retinal damage in hyperglycemic conditions [68].

### 6.2. Macrovascular and Cerebrovascular Protection

Diabetes-associated vascular dysfunction significantly increases the risk of cerebrovascular complications, including ischemic strokes. GS treatment has been shown to reduce blood glucose levels and preserve vascular architecture in experimental models [61].

Structural studies have demonstrated reduced collagen deposition, improved vessel diameter, and preservation of cerebral microvascular integrity. GS also modulates angiogenic signaling pathways, including the suppression of VEGF and Angiopoietin-1 (Ang-1)/Tie-2 signaling, thereby enhancing vascular stability and reducing pathological angiogenesis [61].

### 6.3. Wound Healing, Antimicrobial and Tissue Repair Effects

GS extract significantly accelerated wound healing by enhancing tissue regeneration. Increased activities of antioxidant enzymes, such as SOD, CAT, and glutathione (GSH), along with reduced MDA levels, contribute to decreased oxidative stress and improved fibroblast proliferation and collagen synthesis [63].

GS also exhibits broad-spectrum antimicrobial activity against pathogens, including *Staphylococcus aureus*, *Escherichia coli*, and *Pseudomonas aeruginosa*, as well as antifungal activity against *Candida albicans* [67,69]. These effects are attributed to its rich phytochemical composition, including saponins, flavonoids, and tannins [25,67].

These combined antioxidant and antimicrobial properties support the therapeutic potential of GS in managing diabetic wounds.

### 6.4. Antioxidant and Anti-Inflammatory Effects

GS enhances endogenous antioxidant defense systems and reduces oxidative stress by decreasing lipid peroxidation and increasing the activities of antioxidant enzymes, including SOD, CAT, and GPx [20,21,22].

Experimental studies have demonstrated significant reductions in oxidative stress markers in the serum, liver, and kidney tissues [39,40]. GS also exhibits a strong free radical scavenging capacity, as confirmed by DPPH and ABTS assays [18].

In addition to its antioxidant activity, GS exerts anti-inflammatory effects by suppressing pro-inflammatory cytokines and modulating inflammatory signaling pathways. These effects are closely associated with reduced oxidative stress and improved metabolic balance in the body.

Nanotechnology-based formulations, such as GS-derived zinc nanoparticles, further enhance antioxidant capacity by improving the stability and bioavailability of bioactive compounds [64]. Polyherbal formulations also demonstrate synergistic effects in reducing oxidative stress and inflammation [18,53].

### 6.5. Regulation of Lipid Metabolism

GS plays a significant role in improving lipid metabolism and reducing diabetes-associated dyslipidemia. Experimental and clinical studies have reported reductions in total cholesterol (TC), triglycerides (TG), low-density lipoprotein (LDL), and very-low-density lipoprotein (VLDL) levels, along with increased high-density lipoprotein (HDL) levels [19,58,70].

Mechanistically, GS regulates lipid metabolism by activating lipoprotein lipase (LPL), modulating peroxisome proliferator-activated receptor gamma (PPAR-γ), inhibiting intestinal lipid absorption, and increasing fecal excretion of cholesterol and bile acids [18,25].

Clinical studies and meta-analyses support these findings, although improvements in lipid parameters are often dependent on the treatment duration [8,41].

These effects contribute to improved metabolic homeostasis and reduced cardiovascular risks.

### 6.6. Energy Homeostasis and Body Weight Regulation

GS exhibits dual regulatory effects on body weight and energy metabolism, depending on the metabolic condition. In uncontrolled diabetes, GS attenuates weight loss by improving glucose utilization and reducing protein catabolism, thereby preserving the lean body mass [46].

In obesity-associated conditions, GS improves metabolic balance by modulating lipid metabolism and reducing fat accumulation [42].

Gymnemic acids inhibit intestinal glucose absorption and reduce dietary fat digestion, contributing to reduced body weight and adiposity [18].

Clinical studies have indicated that long-term GS supplementation may reduce body weight and body mass index (BMI), although short-term studies have shown variable results [41,71].

These findings highlight the role of GS in the regulation of appetite, energy balance, and metabolic homeostasis.

Clinical findings further support the therapeutic potential of *G. sylvestre* in the management of diabetes. In a clinical study conducted by Thakur et al. the administration of 1 g/day GS powder reduced fasting blood glucose by 12.9% and postprandial blood glucose by 24.8% in diabetic individuals [11]. Improved glycemic control has also been reported when GS is used in combination with conventional antidiabetic medications.

Additional clinical evidence has demonstrated the potential benefits of GS in different patient groups. A randomized double-blind pilot study reported that GS-supplemented chewing gum reduced sweet food consumption and improved perceived dietary control in individuals with type 1 DM [55]. Similarly, exploratory clinical findings in patients with prediabetes suggested improvements in fasting blood glucose and metabolic parameters following the administration of GS-containing polyherbal formulations [56]. Furthermore, supplementation with GS extract significantly reduced fasting blood glucose levels in pregnant women with a history of gestational DM [57].

These observations were further supported by a meta-analysis conducted by Devangan et al., who evaluated 419 patients and demonstrated significant reductions in fasting blood glucose, postprandial glucose, and HbA1c levels following GS supplementation [8]. However, despite these promising findings, the available evidence remains heterogeneous in terms of experimental design, treatment duration, extract standardization, and outcome measurements. Therefore, large-scale, long-term clinical studies are required to strengthen the translational relevance of GS in diabetes management.

## 7. Translational Strategies and Advanced Therapeutic Approaches of *Gymnema sylvestre*

**Figure 7:**
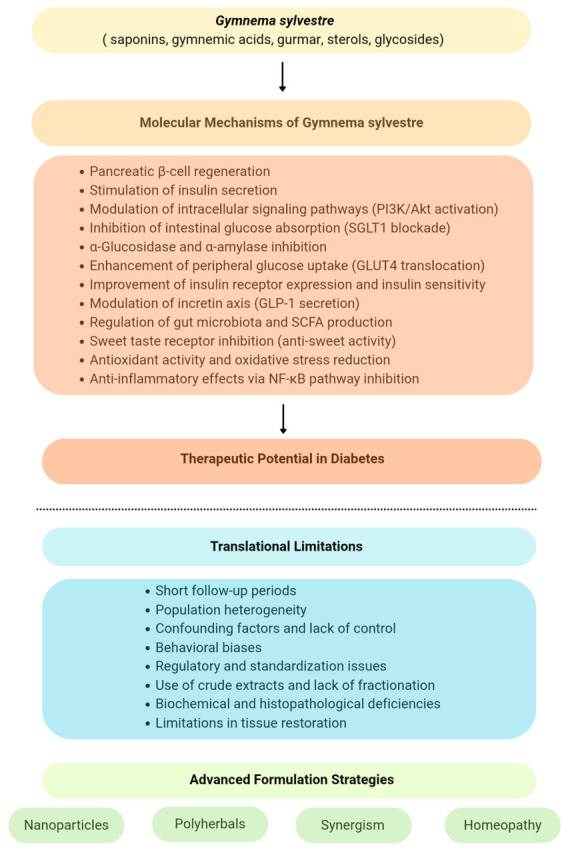
Translational challenges and advanced formulation strategies for *Gymnema sylvestre*. The schematic illustrates the key barriers limiting clinical translation, including phytochemical heterogeneity, lack of standardization, and poor bioavailability of bioactive compounds. It also highlights emerging approaches, such as nanocarriers, phytosomes, nanoemulsions, and controlled-release systems, designed to enhance stability, absorption, and targeted delivery. Together, these strategies aim (Dashed line; separates therapeutic potential from translational limitations). This figure was generated using the DALL-E 3 image generation tool integrated into ChatGPT (OpenAI) [https://chat.openai.com/, accessed on 19 March 2026].

Although numerous experimental and clinical studies support the antidiabetic potential of *G. sylvestre* extract, its clinical application remains limited. The main reasons for this include the low bioavailability of its bioactive compounds, variability in the extract composition, and challenges in delivering adequate amounts to the target tissues. Therefore, recent studies have focused on the development of formulation strategies and investigation of approaches that could enhance therapeutic efficacy (Figure 7).

### 7.1. Nanotechnological Carrier Systems

One of the limitations of the clinical use of GS is the low bioavailability of its bioactive components. Therefore, nanotechnological carrier systems have attracted attention as potential tools for enhancing stability, controlling release, and improving delivery to target tissues.

Daisy et al. developed a graphene oxide-based nanocomposite scaffold by incorporating GS extract and curcumin into a polymeric matrix composed of poly (hydroxybutyrate) and sodium alginate. SEM analysis revealed a porous nano-microstructure that could facilitate oxygen diffusion and support the controlled release of active compounds. This structure also provides a suitable environment for fibroblast adhesion and proliferation, thereby contributing to wound healing [54].

In addition, environmentally friendly nanoparticle synthesis has been investigated. Verma et al. reported the synthesis of zinc nanoparticles using GS leaf extract. These nanoparticles showed increased interactions with key enzymes, such as α-amylase and α-glucosidase, demonstrating enhanced enzyme inhibition compared to crude extracts [64].

Furthermore, recent studies have demonstrated that GS-mediated nanomaterials may exert potent antidiabetic effects that extend beyond those of conventional extracts. Chavan et al. reported the green synthesis of silver nanoparticles (Ag-NPs) using aqueous and alcoholic extracts of *G. sylvestre*. Physicochemical analyses confirmed the formation of spherical and highly stable nanoparticles with significant α-amylase inhibitory activity, exceeding that of acarbose. FTIR findings suggested that phytochemicals such as flavonoids, phenolics, and terpenoids participate in the nanoparticle reduction and stabilization processes. These findings indicate that GS-derived nanoparticles may serve as environmentally friendly and biologically active therapeutic systems for the management of diabetes [72].

In addition to metallic nanoparticles, polymeric nanoformulations have been investigated to improve the oral bioavailability and therapeutic performance of GS bioactive compounds. Rahman et al. developed β-cyclodextrin-based stimuli-responsive nanogels encapsulating *G. sylvestre* extract. The optimized nanoformulation exhibited a high encapsulation efficiency, favorable zeta potential, pH-responsive release behavior, and markedly improved aqueous solubility. In streptozotocin-induced diabetic rats, the nanogel formulation significantly reduced blood glucose levels, enhanced insulin secretion, improved antioxidant defense systems, and ameliorated lipid abnormalities in the rats. Histopathological findings further confirmed pancreatic β-cell regeneration and hepatic tissue repair, suggesting that nanoformulations may substantially improve the translational potential of GS-based therapy [73].

### 7.2. Polyherbal and Synergistic Therapeutic Approaches

Polyherbal formulations are widely used in phytotherapy to achieve broad therapeutic effects. The combination of GS with other medicinal plants may enhance its efficacy through synergistic interactions [74].

For example, Le et al. demonstrated that a combination of Phyllanthus amarus and GS exhibited a stronger α-glucosidase inhibitory effect than GS alone [74].

Similarly, Kousar et al. reported that the combination of GS with Caesalpinia bonduc increased the expression of genes related to β-cell function (Pdx-1, Ins-1, and Ins-2) and suppressed components of the JNK signaling pathway. Histological findings also indicate improvements in islet structure [14].

Additionally, combined herbal treatments have been shown to strengthen antioxidant defense mechanisms via activation of the Keap-1/Nrf-2 pathway, leading to a decrease in MDA levels and an increase in SOD and CAT activity [14].

Combination therapy may also improve metabolic parameters. For instance, the combination of Trigonella foenum-graecum and GS reduced serum urea and creatinine levels and improved liver enzyme profile. A randomized clinical trial of a formulation containing GS and other bioactive components also reported improvements in lipid profiles [75].

Several clinical and experimental studies have further supported the synergistic therapeutic potential of GS-containing polyherbal formulations. Mahajan et al. evaluated the traditional Ayurvedic formulation “GSPF Kwath” in patients with type 2 DM and reported significant reductions in fasting and postprandial glucose levels, HbA1c, total cholesterol, triglycerides, LDL, and VLDL levels following long-term treatment. Improvements in antioxidant defense systems, including glutathione, superoxide dismutase, and catalase activity, were also observed without evidence of hepatic or renal toxicity [76].

Similarly, Shanmugam et al. developed a novel polyherbal aqueous formulation containing *G. sylvestre*, *Momordica charantia*, *Syzygium cumini*, and *Mangifera indica*. The formulation normalized blood glucose levels more effectively than glibenclamide or GS extract alone in streptozotocin-induced diabetic rats. Histopathological analyses demonstrated the restoration of pancreatic islet architecture and regeneration of β-cell morphology, suggesting strong synergistic interactions among the phytochemical constituents [77].

Kurian et al. also investigated the polyherbal formulation “G-400,” which contains *G. sylvestre* together with *Salacia oblonga*, *Tinospora cordifolia*, *Emblica officinalis*, and *Curcuma longa*. Both preclinical and clinical findings have demonstrated improvements in fasting glucose, postprandial glucose, HbA1c, insulin secretion, and lipid profiles without evidence of organ toxicity [78].

Recently, Gopiratnam et al. reported that a standardized polyherbal formulation containing *G. sylvestre*, *Momordica charantia*, *Trigonella foenum-graecum*, *Ocimum sanctum*, and *Azadirachta indica* exhibited strong antihyperglycemic activity, along with favorable pharmaceutical stability and industrial applicability [79]. Collectively, these findings suggest that GS-based polyherbal combinations may provide multi-target metabolic regulation through complementary and synergistic mechanisms.

### 7.3. Modern Formulations and Commercial Applications

In addition to its traditional uses, GS has been incorporated into modern pharmaceutical and nutraceutical products. Glycemic control is offered in various forms, such as tea, dietary supplements, and functional food products.

Some formulations have been developed on an industrial scale. For example, IME-9 and BGR-34 are Ayurvedic formulations that include GS as an active ingredient [25].

These products represent efforts to integrate traditional herbal knowledge with modern pharmaceutical methods.

## 8. Conclusions and Future Perspectives

*G. sylvestre* represents a promising plant-derived therapeutic agent with multifaceted antidiabetic effects supported by experimental and clinical evidence. However, its clinical translation remains constrained by several key limitations, including phytochemical heterogeneity, lack of standardized formulations, variable pharmacokinetic profiles, and insufficient long-term clinical data [15,43].

Taken together, the findings from in vitro, in vivo, and clinical studies suggest that the biological effects of *G. sylvestre* may be complementary across different experimental models. Mechanistic insights obtained from in vitro studies appear to be partially supported by findings from in vivo and clinical investigations, particularly regarding glycemic control and insulin regulation. However, despite these promising observations, clinical evidence remains limited and heterogeneous. Therefore, factors such as bioavailability, metabolic transformation, and variability in the study design should be carefully considered when interpreting the translational relevance of these findings.

To facilitate its effective integration into clinical practice, future research should prioritize the isolation, characterization, and standardization of specific bioactive compounds rather than crude extracts. A detailed investigation of triterpene glycosides that interact with target proteins involved in α-amylase, α-glucosidase, and insulin signaling pathways will provide clearer insights into the underlying mechanisms of action [48,80].

Advanced formulation strategies designed to overcome pharmacokinetic limitations represent a promising approach. Drug delivery systems, such as liposomes, polymeric nanoparticles, phytosomes, nanoemulsions, nanogels, and controlled-release platforms, have the potential to enhance the stability, bioavailability, and targeted delivery of GS bioactives [81,82].

Notably, nanocomposite systems and β-cyclodextrin-based nanogels have been shown to improve therapeutic outcomes, including enhanced wound healing and hypoglycemic effects, primarily by improving the solubility, stability, and controlled release of *G. sylvestre* bioactive compounds [54,83].

In summary, overcoming these translational barriers will require a multidisciplinary approach integrating phytochemistry, pharmacology, and clinical sciences. The combination of standardized *G. sylvestre* extracts with advanced delivery technologies represents a promising and potentially effective strategy to effectively bridge the gap between experimental research and clinical applications. Future research should focus on well-designed randomized controlled trials, standardized extract formulations, and detailed pharmacokinetic analyses. Additionally, innovative approaches, such as nanotechnology-based delivery systems and multi-component phytotherapy strategies, may enhance the therapeutic potential of *G. sylvestre*. In conclusion, although *G. sylvestre* shows significant promise as a multi-targeted antidiabetic agent, a more holistic, critical, and transformative research approach is required to fully establish its clinical relevance.

## Figures and Tables

**Table 1:** Major Phytochemical Constituents of *Gymnema sylvestre* and Their Antidiabetic Relevance.

Phytochemical Group	Representative Compounds	Primary Biological Activities	Relevance to Diabetes	References
Triterpenoid saponins (major group)	Gymnemic acids (I–XVIII), Gymnemagenin, Gymnemosides	Inhibition of intestinal glucose absorption; stimulation of insulin secretion; β-cell protective effects	Key antidiabetic constituents; reduce postprandial glucose and improve glycemic control	[10,15,17,29]
Peptide compounds	Gurmarin and gurmarin-like peptides (Gur-1–Gur-9)	Inhibition of sweet taste receptor (T1R2/T1R3)	May reduce sweet taste perception and indirectly support glycemic control	[12,23,30]
Flavonoids	Kaempferol derivatives and flavonol glycosides	Antioxidant and anti-inflammatory activities	Associated with reduction of oxidative stress linked to diabetic complications	[13]
Plant sterols and triterpenes	Stigmasterol, Lupeol, β-amyrin	Anti-inflammatory and bioactive properties	Metabolic support	[9,11]
Fatty acids and other minor phytochemicals	n-Hexadecanoic acid, phytol, squalene	Bioactive phytochemical constituents	May contribute to supportive metabolic effects	[27,31]
Mineral elements	Zn, Mg, Ca, Fe, K	Cofactors in metabolic enzyme systems	Support glucose metabolism and insulin-related function	[25,32]

**Table 2:** Summary of experimental and clinical studies on *G. Sylvestre*. ↑: increase; ↓: decrease; ↔: no change.

Preparation	Experimental Model	Dose	Effect
*G. sylvestre* extract chewing gum [55]	Adult T1DM patients using CGM reporting frequent sweet intake	169–210 mg/kg	Sweet food consumption ↓ Glucose ↓
Homeopathic mother tinctures (MTs) derived from *G. sylvestre, Cephalandra indica*, or *Syzygium jambolanum* [56]	Patients diagnosed with prediabetes	Patient-specific individualized dosages	Glucose ↓
*G. sylvestre* extract [57]	2nd or 3rd-trimester pregnant women with a history of gestational diabetes	200 mg/kg	Fasting glucose ↓
Various *G. sylvestre* extracts [8]	T2DM patients (*n* = 419)		Fasting & postprandial glucose ↓ HbA1c ↓ Total cholesterol & triglycerides ↓
*G. sylvestre* supplementation [58]	Adult patients diagnosed with prediabetes	600 mg/kg	Insulin sensitivity ↑ BMI & LDL ↓ HbA1c ↓
*G. sylvestre* dried leaf powder [41]	T2DM patients (aged 30–60, Nepal, *n* = 40)	6 g/day	Fasting & postprandial glucose ↓
Om Santal Adivasi (OSA^®^) [45]	T2DM patients and Isolated human pancreatic islets (Islets of Langerhans/Beta cells)	1 g/kg	Fasting & postprandial glucose ↓ Insulin & C-peptide ↑
*G. sylvestre* extract [49]	Drug-naïve patients aged 30–60 diagnosed with metabolic syndrome, *n* = 24	600 mg total (fixed daily dose)	BMI &VLDL ↓
*G. sylvestre* leaf extract [5]	STZ-induced Wistar rats		Nerve tissue damage ↓ Hyperalgesia ↓
*G. sylvestre* aqueous leaf extract (ALGS) [59]	T2DM male Sprague-Dawley rats	0.5% and 1% (*w*/*w*)	Glucose, serum creatinine, & urea nitrogen ↓ SOD, CAT, GPx, & insulin ↑ Renoprotective effect ↑
*G. sylvestre* aqueous and methanolic extracts [60]	T2DM Wistar rats	100 (Aqueous extract)	Serum ALT, AST ↓
*G. sylvestre* aqueous and methanolic extracts [60]	T2DM Wistar rats	7.5 (Methanolic extract and fractions)	Renal mesangial expansion ↓ Body and liver weights ↔
Gymnemic acid (>75% purity) [50]	T2DM male Wistar rats	400 mg/kg	Glucose, BUN & creatinine ↓ Renal segmental & interlobar artery diameters ↑ Arterial wall VEGF expression ↓
*G. sylvestre* aqueous (Aq) (ethanolic) [37]	STZ-induced Wistar albino rats	50,100 mg/kg	Blood glucose & lipid ↓ Antioxidant enzyme ↑ Neuroprotective effect
Gymnemic acid (>75% purity) [61]	STZ-induced male Wistar rats	400 mg/kg	Glucose ↓ Brain tissue VEGF & Angiopoietin-1 expression ↓ Cerebral vascular repair & vessel diameter dilation ↑
Gymnemic acid [62]	Diabetic male Wistar rats	400 mg/kg	Preservation of choriocapillaris structure ↑ Choroidal VEGF & CD31 expression ↓ Choroidal layer & arterial wall thickness ↓
*G. sylvestre* aqueous (Aq) and methanolic (MeOH) extracts [63]	Normoglycemic female Wistar rats	10% ointment	Wound closure rate in normoglycemic rats ↑
*G. sylvestre* aqueous (Aq) and methanolic (MeOH) extracts [63]	Dexamethasone-induced male rats	(Aq) 7.5 mg/kg (MeOH) mg/kg	MDA ↓ CAT, SOD &GSH ↑
*G. sylvestre* leaf extracts and gymnemic acid [18]	Type 1 and type 2 diabetic animal models	Various doses (Generally 200–400)	Insulin secretion ↑ Intestinal glucose absorption ↓ Body weight & lipid accumulation ↓
*G. sylvestre* hydroalcoholic extract (HAGS) [22]	STZ-induced male Wistar rats	100, 200, and 400 mg/kg	IR gene expression ↑ Proglucagon gene expression & GLP-1 secretion ↑ Glucose & HbA1c ↓ Insulin, SOD & CAT ↑
Polyherbal formulation (Ethanolic extracts containing *G. sylvestre leaves*, *Cinnamomum zeylanicum bark*, *Eugenia jambolana* seeds, and *Vinca rosea*) [53]	STZ-induced Wistar rats	400 mg/kg	Blood glucose and lipid ↓ Total cholesterol, triglyceride, & LDL ↓ HDL ↑ SOD & CAT ↑
Crude methanolic extract and fractions (N-hexane, Ethyl acetate, N-butanol, Aqueous) [64]	Alloxan-induced Rattus norvegicus	100, 300, and 600 mg/kg	Glucose ↓ Total cholesterol, triglycerides & LDL ↓ HDL ↑ Maintenance of body weight
*G. sylvestre* aqueous (Aq) Ethanol Extract [19]	STZ-induced Sprague-Dawley rats	125, 250, and 500 mg/kg	Regulates intestinal microbiota SCFA production ↑ Total cholesterol, triglycerides & LDL-C ↓ HDL-C ↑ Fasting glucose ↓ Suppresses body weight gain
Hydroalcoholic extract of *G. sylvestre* leaves [65]	High-cholesterol diet-induced hyperlipidemic female Sprague-Dawley rats	200 mg/kg	Total cholesterol, triglycerides, LDL & VLDL ↓ HDL ↑
70% Ethanolic *G. sylvestre* leaf extract [46]	Alloxan-induced	500, 1000, and 1500 mg/kg	Glucose ↓ Total cholesterol, triglycerides & LDL ↓ HDL ↑ SGOT, SGPT, urea & creatinine ↓
Polyherbal formulation (containing *G. sylvestre*, *Syzygium cumini*, *Momordica charantia*, *Emblica officinalis*, *Enicostemma littorale*, *Azadirachta indica*, *Tinospora cordifolia*, and *Curcuma longa*) [34]	STZ-induced rats	100 mg/kg	Total cholesterol, triglycerides, LDL & VLDL ↓ Glucose ↓ Insulin ↑ Blood urea & creatinine ↓ GSH, SOD & CAT ↑
*G. sylvestre* leaf extracts (GS3, GS4) [35]	STZ-induced Wistar albino rats	Oral, repeated dosing	Glucose homeostasis ↑ Insulin secretion ↑
70% ethanol hydroalcoholic extract of *G. sylvestre* (and other plants in the study: *Azadirachta indica*, *Catharanthus roseus*, and *Ocimum sanctum*) leaves [36]	STZ-induced Swiss albino rats	50, 100, 200, and 400 mg/kg	Glucose ↓
*G. sylvestre* and combined with *Caesalpinia bonduc* [14]	Alloxan-induced Wistar rats	Single: 400 mg/kg Combined: 200 mg/kg *G. sylvestre* + 200 mg/kg *C. bonduc*	HbA1C & fasting glucose ↓ SOD & CAT ↑
*G. sylvestre* extract [38]	STZ-induced Wistar albino rats	200–400 mg/kg *G. sylvestre* doses compared with Metformin	GLUT-2 expression ↑ Regeneration of pancreatic beta-cells ↑
*G. sylvestre* leaf powder [40]	Alloxan-induced Wistar albino rats	250, 500 mg/kg	Glucose ↓ Insulin ↑ AST & ALT ↓ Expression of Ins-1, Ins-2, and liver metabolism genes ↑
Crude saponin fraction from the MeOH extract of *G. sylvestre* leaves and five isolated triterpene glycosides [66]	STZ-induced mice	60 mg/kg	Except for gymnemic acid IV, the other 4 glycosides showed no effect Glucose ↓
Methanolic *G. sylvestre* leaf extract [67]	Klebsiella pneumoniae, Staphylococcus aureus, Pseudomonas aeruginosa, and Escherichia coli strains	0.01–1.0 mg/mL	Antibacterial activity
Om Santal Adivasi (OSA^®^) [43]	Mouse-derived MIN6 pancreatic β-cell line and fresh pancreatic islets isolated from mice	Does not contain dose data in mg/kg	Stops apoptosis triggered by IL-1β, TNF-α & IFN-γ

## Data Availability

Not applicable. This review is a synthesis and analysis of published literature and did not generate or analyze new experimental data.
